# Cancer-Derived VEGF-C Increases Chemokine Production in Lymphatic Endothelial Cells to Promote CXCR2-Dependent Cancer Invasion and MDSC Recruitment

**DOI:** 10.3390/cancers11081120

**Published:** 2019-08-06

**Authors:** Jing-Yi Chen, You-Syuan Lai, Pei-Yi Chu, Shih-Hsuan Chan, Lu-Hai Wang, Wen-Chun Hung

**Affiliations:** 1 National Institute of Cancer Research, National Health Research Institutes, Tainan 704, Taiwan; 2 Department of Pathology, Show Chwan Memorial Hospital, Changhua City 500, Taiwan; 3 Chinese Medicine Research Center and Graduate Institute of Integrated Medicine, China Medical University, Taichung 404, Taiwan; 4 Institute of Molecular and Genomic Medicine, National Health Research Institutes, Miaoli 350, Taiwan; 5 Graduate Institute of Medicine, College of Medicine, Kaohsiung Medical University, Kaohsiung 807, Taiwan; 6 Drug Development and Value Creation Research Center, Kaohsiung Medical University, Kaohsiung 807, Taiwan

**Keywords:** vascular endothelial growth factor-C, CXC chemokine receptor 2, lymphovascular niche, myeloid-derived suppressor cells

## Abstract

Breast cancer-derived vascular endothelial growth factor-C (VEGF-C) has been shown to enhance lymphangiogenesis in lymph nodes to accelerate cancer metastasis. However, the remodeling of lymph node microenvironments by VEGF-C remains elusive. By in vivo selection, we established a subline (named as “LC”) with strong lymphatic tropism and high VEGF-C expression from the human MDA-MB-231 breast cancer cell line. Co-culture with LC cells or treatment with LC-conditioned medium upregulated the expression of CXC chemokines in lymphatic endothelial cells (LECs), which could be inhibited by pre-incubation with VEGF-C-neutralizing antibodies and VEGFR3 inhibitors. The chemokines produced by LECs enhanced recruitment of myeloid-derived suppressor cells (MDSCs) to tumor-draining and distant lymph nodes in tumor-bearing mice. Treatment with a CXCR2 inhibitor after tumor cell inoculation dramatically decreased the number of MDSCs in lymph nodes, suggesting the importance of the chemokine/CXCR2 signaling axis in MDSC recruitment. In addition, LEC-released chemokines also stimulated the expression of serum amyloid A1 (SAA1) in cancer cells, enhancing their lymphatic invasion by increasing VE-cadherin phosphorylation, junction disruption, and vascular permeability of LECs. Clinical sample validation confirmed that SAA1 expression was associated with increased lymph node metastasis. Collectively, we reveal a novel mechanism by which cancer cell-derived VEGF-C remodels lymphovascular microenvironments by regulating chemokine production in LECs to promote cancer invasion and MDSC recruitment. Our results also suggest that inhibition of CXCR2 is effective in treating lymphatic metastasis.

## 1. Introduction

The lymphatic system is involved in diverse physiological and pathological processes like immune modulation, tissue fluid homeostasis, wound healing, chronic inflammation, and tumor metastasis. Many cancers disseminate first through lymphatic system, and metastasis in sentinel lymph nodes indicates the spread of tumors from a primary site [1,2]. The presence of tumor cells in sentinel or draining lymph nodes is a major prognostic indicator in breast, melanoma, prostate, and head and neck cancers [3,4,5,6]. Recently, the concept of a passive role of lymphatic endothelial cells (LECs) in cancer metastasis has been challenged. LECs have been shown to release CCL21 and CXCL12 (SDF-1) to recruit CCR7- and CXCR4-expressing cancer cells into sentinel lymph nodes (LNs) [7]. In addition to CCL21 and CXCL12, CCL1 produced from LN lymphatic sinuses also enhances lymph node entry of CCR8-expressing melanoma cells [8]. When the CCR8 signaling pathway is abolished, lymph node metastasis is significantly reduced [8]. However, the role of tumor-associated LECs in the remodeling of the lymphovascular niche is still unclear.

The remodeling of the lymphovascular niche includes two major steps. First, cancer cells release lymphangiogenic factors like VEGF-C and VEGF-D to stimulate proliferation of LECs and increase and enlarge lymphatic vessels, which results in increased lymphatic flow and cancer cell entry. The second step is the recruitment of myeloid-derived suppressor cells (MDSCs) into lymph nodes. MDSCs originate from myeloid progenitor cells in bone marrow and are composed of heterogeneous immature myeloid cells. Various pathological conditions, such as autoimmune disease, infection, and cancer, induce the expansion of MDSCs in bone marrow and the spleen. These cells then migrate into the blood stream and accumulate in lymphoid organs and tumors in cancer patients. MDSCs may inhibit the antitumor immune activities of CD4^+^ T cells, CD8^+^ T cells, and natural killer (NK) cells [9,10,11]. Emerging evidence has suggested that MDSCs mediate T cell tolerance in tumor-draining lymph nodes, and the enrichment of cancer-educated LECs is critical in modulating adaptive immune responses in lymph nodes [12,13]. However, whether the reciprocal interaction between cancer cells and LECs plays a role in the recruitment of MDSCs is currently unknown.

In this study, we investigated the alteration of the lymphovascular niche after implantation of breast cancer cells with differential lymphatic tropisms and VEGF-C expressions into mice. In addition, we tried to elucidate the underlying mechanism by which cancer-educated LECs promote cancer metastasis and MDSC recruitment.

## 2. Materials and Methods

### 2.1. Cell Culture and Reagent

Parental MDA-MB-231 and high lymphatic-tropic metastasis subline cells (named as “LC cells”) were established as previously described [14]. Cells were cultured in Dulbecco’s Modified Eagle Medium with 10% fetal bovine serum. Human dermal LECs were purchased from PromoCell (Heidelberg, Germany) and cultured in MV2 endothelial cell growth medium. Anti-pan-cytokeratin-488 and anti-LYVE-1 antibodies were obtained from Abcam (Cambridge, MA, USA). Anti-mouse NK1.1 antibody was purchased from MyBioSource (San Diego, CA, USA). Anti-SAA1 antibody was purchase from DAKO, Agilent Technologies (Santa Clara, CA, USA). Fluorescein isothiocyanate-conjugated human serum amyloid A1 (SAA1) antibody was obtained from Assaypro LLC (St. Charles, MO, USA). Anti-α-Tubulin antibody was purchased from GeneTex Inc. (Hsinchu, Taiwan). Anti-mouse CD11b and anti-mouse Ly6G/6C antibodies were obtained from BD Transduction Laboratories (San Jose, CA, USA). PE-Cyanine5.5-conjugated anti-mouse Ly6G (Gr-1), Alexa Fluor 488-conjugated anti-mouse CD11b, and anti-phospho-VE-cadherin [Y658] antibodies were purchased from Thermo Fisher Scientific Inc. (Waltham, MA, USA). SAA1 recombinant protein and an ELISA kit were purchased from Abcam (Cambridge, MA, USA). GM-CSF, SDF-1, CXCL5, CCL19, and CCL21 recombinant proteins were obtained from Cell Guidance Systems LLC (St. Louis, MO, USA). SB225002 was purchased from Tocris Bioscience (Minneapolis, MN, USA). IAXO-102 was obtained from Adipogen Corp. (San Diego, CA, USA).

### 2.2. Human Gene Expression Analysis

LECs (1 × 10^5^) were co-cultured with MDA-MB-231 or LC cells (5 × 10^4^) for 72 h, and total RNAs were isolated using an RNA extraction kit (Geneaid, New Taipei City, Taiwan). RNAs were converted to double-stranded cDNA and amplified using in vitro transcription with T7 polymerase. DNA microarray analysis was performed by using the Human OneArray v6 (Phalanx Biotech, Hsinchu, Taiwan). Data were analyzed with Rosetta Resolver System software (Rosetta Biosoftware, USA). Pathway analysis was analyzed with GSEA software.

### 2.3. RNA Extraction and Quantitative Reverse Transcription-PCR Analysis

Total RNA was isolated from cells and 1 μg of RNA was reverse-transcribed to cDNA with MMLV reverse transcriptase (Promega). Target mRNAs were quantified using real-time PCR reactions with SYBR green fluorescein, and actin was served as an internal control. cDNA synthesis was performed at 95 °C for 5 min, and the conditions for PCR were 30 cycles of denaturation (95 °C/45 s), annealing (60 °C/45 s), extension (72 °C/45 s), and one cycle of final extension (72 °C/10 min). The primer sequence of the target gene is listed in Supplementary Table S1.

### 2.4. Western Blotting

Cellular proteins were extracted from LC cells or LECs with RIPA buffer (50 mM Tris-HCl, pH 7.4, 150 mM NaCl, 1% NP-40, 0.1% sodium dodecyl sulfate (SDS), 0.5% sodium deoxycholate, 2 mM EDTA, and 50 mM NaF), and the proteins were separated by SDS-polyacrylamide gel electrophoresis. Proteins were transferred to poly-vinylidene fluoride membranes, probed with various primary antibodies, and developed by enhanced chemiluminescence reagents as previously described [15].

### 2.5. Quantification of SAA1 by ELISA Assay

LECs (1 × 10^5^) were co-cultured with or without LC cells (5 × 10^4^) for 72 h in a Transwell unit. After co-culturing, the supernatant of the LECs was harvested, and the concentration of SAA1 was investigated using a SAA1 ELISA kit according to the manufacturer’s procedure. Briefly, 100 µL of the supernatant and standard were added to an antibody-precoated microplate overnight at 4 °C. After washing, biotinylated SAA1 antibody and horse reddish peroxidase-streptavidin solution were added sequentially. Finally, the reaction was developed by TMB One-Step Substrate Reagent and was terminated by stop solution. The optical density (O.D.) at 450 nm was read using a microplate reader. The concentration of SAA1 in the medium was calculated by the standard curve and was normalized with the cell protein concentration.

### 2.6. In Vivo Orthotopic Animal Study

LC cells (2 × 10^6^) were suspended in Hank’s balanced salt solution and inoculated into the fourth mammary fat pads of 6 week-old female BALB/cAnN.Cg–Foxn1nu/CrlNarl mice. After one week, mice were treated with CXCR2 antagonist SB225002 (1 mg/kg) via intraperitoneal administration five times per week. Measurement of tumor growth was begun one week after injection, and tumor volume was calculated using the equation: Tumor volume = (length × width^2^)/2. Tumors were harvested from mice three weeks after SB225002 treatment. Tumor weights were measured and statistical differences between various experimental groups were evaluated by *t*-test. Animal use protocol was approved by the Institutional Animal Care and Use Committee of National Health Research Institutes (ethic code: NHRI-IACUC-103153-A) on 2 February 2015.

### 2.7. Flow Cytometric Analysis of CD11b^+^/Gr1^+^ MDSC Population

Cells were isolated from mouse lymph nodes and tumors, and incubated with anti-CD11b and anti-Gr1 antibodies for 1 h at 4 °C. Cells were washed and incubated with Alexa Fluor 594-conjugated anti-rabbit and Alexa Fluor 488-conjugated anti-rat IgG antibodies for another 1 h. The CD11b^+^/Gr1^+^ cells were detected by flow cytometry.

### 2.8. LEC Permeability Assay

LECs (1 × 10^5^) were seeded onto the Transwell upper chamber (pore size: 1 μm) and cultured for 48 h to form a confluent monolayer on the membrane. LECs were treated with SAA1 (50 ng/mL) in the absence or presence of IAXO-102 for 12 h. After treatment, the SAA1-containing medium was removed and fresh serum-free medium with 1 mg/mL FITC–dextran was added to the upper chamber. After 60 min, 50 μL of medium was taken from the lower chamber and the amount of FITC–dextran diffused into the lower chamber was detected by a fluorometer (excitation 485 nm, emission 520 nm). The amount of FITC–dextran detected in the lower chamber reflected the permeability of LEC monolayer.

### 2.9. Immunofluorescent Staining

The membrane of the Transwell unit with the LEC monolayer was also collected for immunofluorescent staining. LECs on the membrane were fixed with 3.7% formaldehyde for 15 min at room temperature. After PBS washing, LECs were permeabilized by 0.1% Triton X-100 solution for 10 min and were incubated with 0.05% bovine serum albumin solution to block nonspecific binding. Anti-VE-cadherin antibody was applied and incubated at room temperature for 1 h. After extensive washing, Alexas Fluro 488 anti-mouse IgG was added and incubated for another 1 h. Finally, the membranes were washed twice with PBS and placed in mounting solution. The monolayer structure of LECs was observed under a fluorescent microscope.

### 2.10. Tissue Microarray

SAA1 expression in human breast cancer was analyzed in two breast tissue microarrays (BC081120a and BRM961) purchased from US Biomax. The BC081120a microarray contained 100 cases of invasive ductal carcinoma and 10 adjacent breast tissues. The breast carcinoma metastatic tissue microarray BRM961 contained 48 cases of primary breast tumor, of which 36 matched metastatic breast tumors in lymph nodes and 12 matched normal tissues. Immunohistochemistry was conducted to examine SAA1 expression in the tissue microarray, and the SAA1 staining intensity was analyzed using HistoQuest image analysis software.

### 2.11. Staining of Mouse Tissues

Tumor and lymph node tissues collected from mice were fixed in neutral-buffered 10% formalin solution, embedded in paraffin, and sectioned into 3 μm slides. Primary antibodies were applied and incubated at 4 °C overnight. After washing, the sections were incubated with HRP-conjugated secondary antibody for 30 min at room temperature. Finally, the sections were counterstained with hematoxylin and observed under a Leica DM2000 microscope (Leica microsystem, Washington, DC, USA). For the detection of NK cells, tumor and lymph node tissues were stained with Alexa Fluor 488-conjugated anti-pan cytokeratin (pan-CK) and anti-NK1.1 antibodies, and the sections were counterstained with 4′,6-diamidino-2-phenylindole (DAPI). The slides were observed under a fluorescent microscope.

### 2.12. Statistical Analysis

Statistical comparisons were performed using Student’s unpaired *t*-tests to investigate the differences in different experimental groups. Data were expressed as the mean ± SEM, and *p* < 0.05 was considered statistically significant. Data analysis was performed using the GraphPad Prism version 5.01 (GraphPad Software, Inc., San Diego, CA, USA).

## 3. Results

### 3.1. Lymphatic-Tropic LC Breast Cancer Cells Derived from MDA-MB-231 Cells Exhibit Strong Lymphatic Invasion Activity

**Figure 1:**
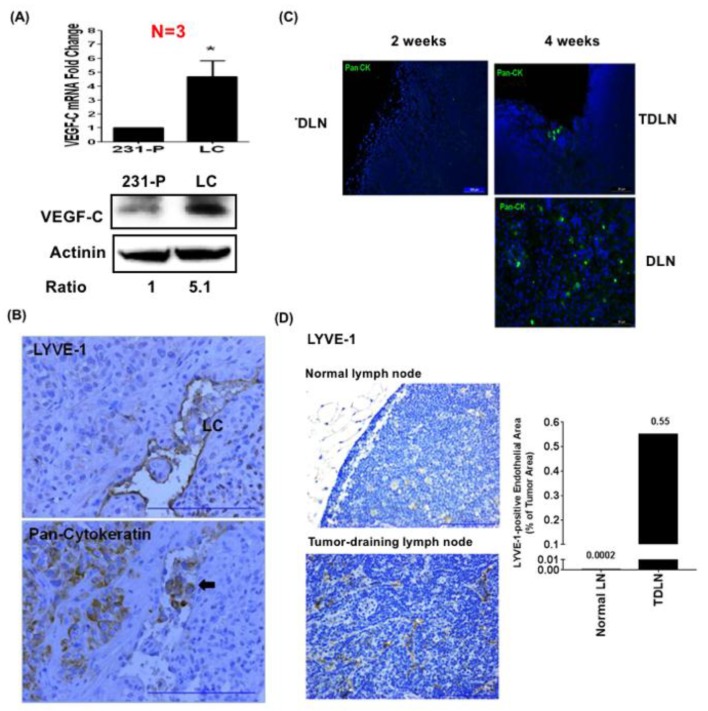
High lymphatic-tropic metastasis LC cells derived from MDA-MB-231 cells via in vivo selection express high levels of VEGF-C and show strong lymphatic invasion ability. (**A**) Quantitative RT-PCR assay demonstrated a 4.7-fold increase of VEGF-C expression in LC cells when compared with parental MDA-MB-231 cells (231-P). VEGF-C protein levels were also increased in LC cells. * *p* < 0.05. (**B**) The tumors generated from LC cells showed intensive lymphanagiogenesis (upper panel) as evidenced by LYVE-1-positive lymphatic endothelial vessels. The invasion of LC cancer cells was also shown by the pan-cytokeratin-positive cells in the lumens of lymphatic vessels. (**C**) Immunofluorescent staining demonstrated the appearance of LC cancer cells in tumor-draining lymph nodes (TDLN) and distant lymph nodes (DLN). Scale bar: 50 μm. (**D**) The TDLN of LC tumors isolated at week four after cancer cell inoculation showed extensive lymphangiogenesis, while very little lymphangiogenesis was found in the lymph nodes of normal mice. Scale bar: 50 μm.

To understand how breast cancer cells modulate lymph node microenvironments to promote tumor metastasis, we injected MDA-MB-231 breast cancer cells into the mammary fat pads of nude mice and established a lymphatic-tropic LC cell line via in vivo selection [14]. We found that LC cells showed a 4.7-fold upregulation in the expression of VEGF-C (Figure 1A). In addition, the increase of the VEGF-C protein level was evidenced by Western blotting (Figure 1A). Immunohistochemical staining demonstrated the increase of lymphatic vessels (as evidenced by LYVE-1-positive endothelial cells) in the tumors generated by LC cells in mice (Figure 1B). In addition, we also found the invasion of cancer cells (confirmed by pan-cytokeratin-positive cells) into the lymphatic vessels in the tumors (Figure 1B). Immunofluorescent staining demonstrated the presence of cancer cells in tumor-draining and distant lymph nodes in LC tumor-bearing mice (4 weeks after injection of LC cells) (Figure 1C). Very little lymphangiogenesis was found in the lymph nodes of normal mice (Figure 1D). On the contrary, the isolated lymph nodes from mice injected with LC cells showed intensive lymphangiogenesis, as shown by the increase of LYVE-1-positive lymphatic vessels (Figure 1D). These data suggested that LCs exhibited strong lymphatic tropism, and this cell line could be a useful model for the study of cancer-modulated lymphovascular niches.

### 3.2. LC Cells Induce an Inflamed Lymphovascular Signature in LECs via VEGF-C

**Figure 2:**
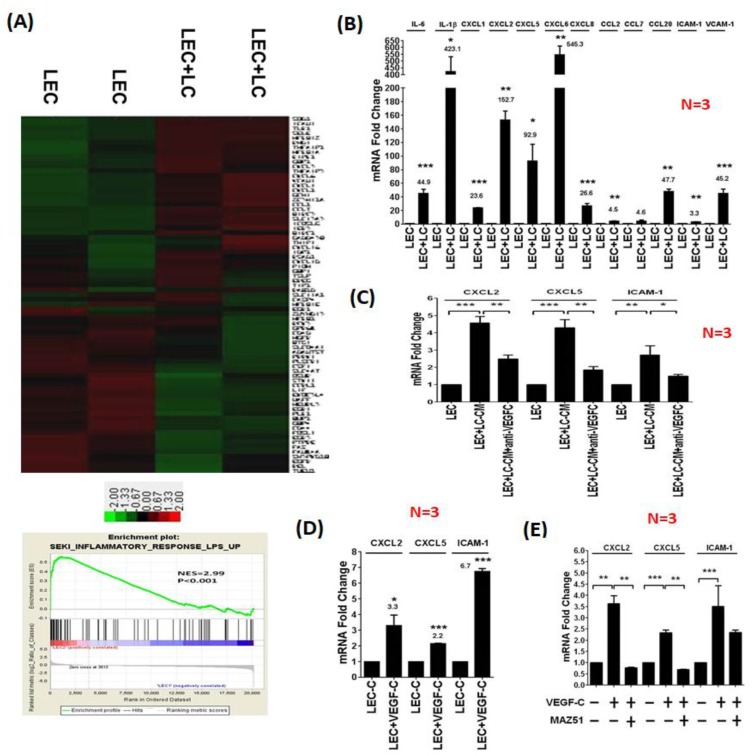
VEGF-C released from LC cancer cells transforms naive lymphatic endothelial cells (LECs) into inflamed LECs. (**A**) LECs were co-cultured with LC cells for 2 days, and the total RNA of the LECs was harvested for microarray study. The heatmap demonstrated the alteration of gene expression in LECs co-cultured with LC cells. Gene set enrichment assay (GSEA) showed the increase of inflammatory genes and chemokines in LECs co-cultured with LC cells. (**B**) Quantitative RT-PCR assay confirmed the increase of upregulated genes identified in the microarray study. (**C**) Depletion of VEGF-C by antibody reduced the increases of CXCL2, CXCL5, and ICAM-1 expression induced by co-culture with LC cells, indicating that VEGF-C plays a crucial role in the transformation of naive LECs into inflamed LECs. (**D**) Treatment with VEGF-C recombinant protein also upregulated inflammatory genes and chemokines in naive LECs. (**E**) Inhibition of VEGFR3 by a specific inhibitor, MAZ51, in LECs significantly decreased the increases of CXCL2, CXCL5, and ICAM-1 expression induced by co-culture with LC cells, indicating that cancer-derived VEGF-C stimulates these genes via VEGFR3 in LECs. * *p* < 0.05; ** *p* < 0.01; *** *p* < 0.001.

We co-cultured LECs with LC cells and harvested RNAs from LECs for microarray analysis. Gene set enrichment assay (GSEA) analysis demonstrated that co-culturing with LC cells significantly upregulated the expression of chemokines and inflammatory genes in LECs, suggesting that LC cells transformed naïve LECs into inflamed LECs (Figure 2A). Interestingly, several pathways including cell cycle checkpoint, DNA repair, adipogenesis, and lipid metabolism in LECs were down-regulated after co-culturing with LC breast cancer cells. The biological significance of these pathways in the crosstalk between cancer cells and LECs needs further characterization. We specifically focused on the effect of LC cell-induced chemokine expression in LECs because chemokines have been shown to play a crucial role in promoting tumor metastasis. We validated the microarray results by quantitative reverse transcription-PCR analysis and confirmed the increases of inflammatory molecules (IL-6 and IL-1β), adhesion molecules (ICAM-1 and VCAM-1), and CXC chemokines (CXCL1, CXCL2, CXCL5, and CXCL8) (Figure 2B). In addition, regulatory T cells (Treg)-trafficking chemokine CCL20 was upregulated in LECs co-cultured with LC cells. Similar gene expression profiles were found in LECs stimulated with the condition medium from LC cells (Supplementary Figure S1). Since tumor-derived VEGF-C controlled the proliferation, survival, and migration of LECs, we investigated whether VEGF-C contributed to the formation of inflamed LECs. When VEGF-C was depleted from the condition medium of LC cells with VEGF-C antibodies, the expressions of CXCL2, CXCL5, and ICAM-1 were significantly decreased (Figure 2C). Treatment with recombinant VEGF-C upregulated the expression levels of CXCL2, CXCL5, and ICAM-1 by 3.3-, 2.1- and 6.7-fold in LECs, respectively (Figure 2D). Pre-incubation of the VEGFR3 inhibitor MAZ-51 significantly reduced the VEGF-C-increased levels of CXCL2 and CXCL5 in LECs (Figure 2E). These data suggested that LC cells modulated gene expression in LECs via the VEGF-C/VEGFR3 axis and transformed naive LECs to inflamed LECs to create an enriched lymphovascular microenvironment.

### 3.3. LEC-Derived Chemokines Promote Lymph Node Metastasis and Lymphangiogenesis

**Figure 3:**
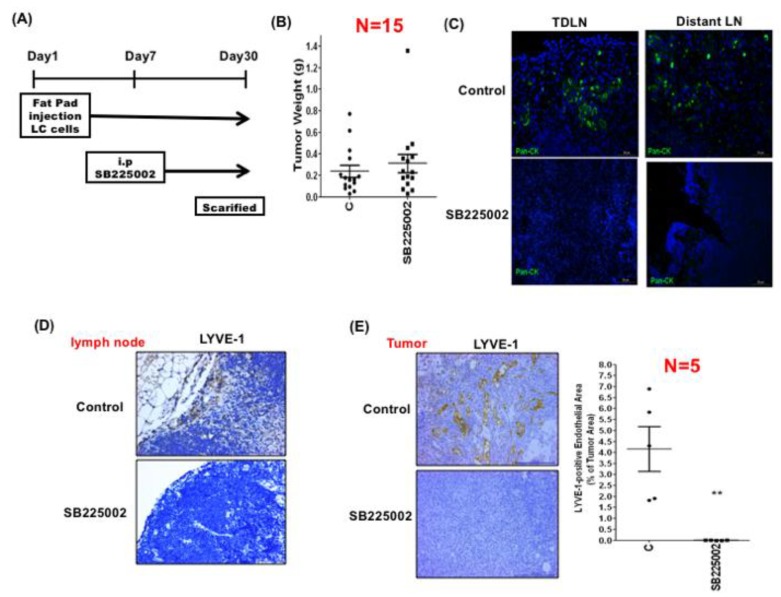
LEC-released chemokines promote cancer cell invasion and lymphangiogenesis in the lymph nodes via CXCR2. (**A**) LC cells were injected into the mammary fat pads of nude mice. After one week, mice were treated with vehicle (control) or CXCR2 inhibitor SB225002 for another three weeks. The tumors and lymph nodes were harvested for analysis. (**B**) The tumor weights of the control and SB225002-treated mice were not significantly different. (**C**) Immunofluorescent staining demonstrated that the number of pan-cytokeratin-positive cancer cells was significantly decreased in the SB225002-treated mice, suggesting that inhibition of CXCR2 reduced the invasion of cancer cells to lymph nodes. (**D**) Lymphangiogenesis in the tumor-draining lymph nodes was suppressed by SB225002. Scale bar: 50 μm. (**E**) Lymphangiogenesis in the tumors was also inhibited by SB225002. Scale bar: 50 μm. ** *p* < 0.01.

The chemokines (CXCL1, 2, 5, and 8) upregulated by cancer-derived VEGF-C in LECs are all cognate ligands of CXCR2. We therefore investigated whether these chemokines affected tumor growth and lymphatic invasion via CXCR2. Both MDA-MB-231 and LC breast cancer cells expressed CXCR2, and the CXCR2 levels were higher in LC cells (Supplementary Figure S2). LC cells were inoculated into mouse mammary fat pads. After one week, tumor-bearing mice were treated without or with CXCR2 inhibitor SB225002 for another 3 weeks (Figure 3A). SB225002 did not affect primary tumor growth (Figure 3B). However, metastatic cancer cells in tumor-draining and distant lymph nodes detected by pan-cytokeratin staining were significantly reduced in SB225002-treated mice (Figure 3C). In addition, lymphangiogenesis, detected by LYVE-1-positive lymphatic vessels in the lymph nodes, was also reduced after SB225002 treatment (Figure 3D). Interestingly, we found that lymphangiogenesis was also decreased in primary tumors (Figure 3E). These data indicated that chemokines released from LECs could promote lymph node invasion and lymphangiogenesis via CXCR2.

### 3.4. LEC-Derived Chemokines Promote the Recruitment of MDSCs to Tumors and Lymph Nodes

**Figure 4:**
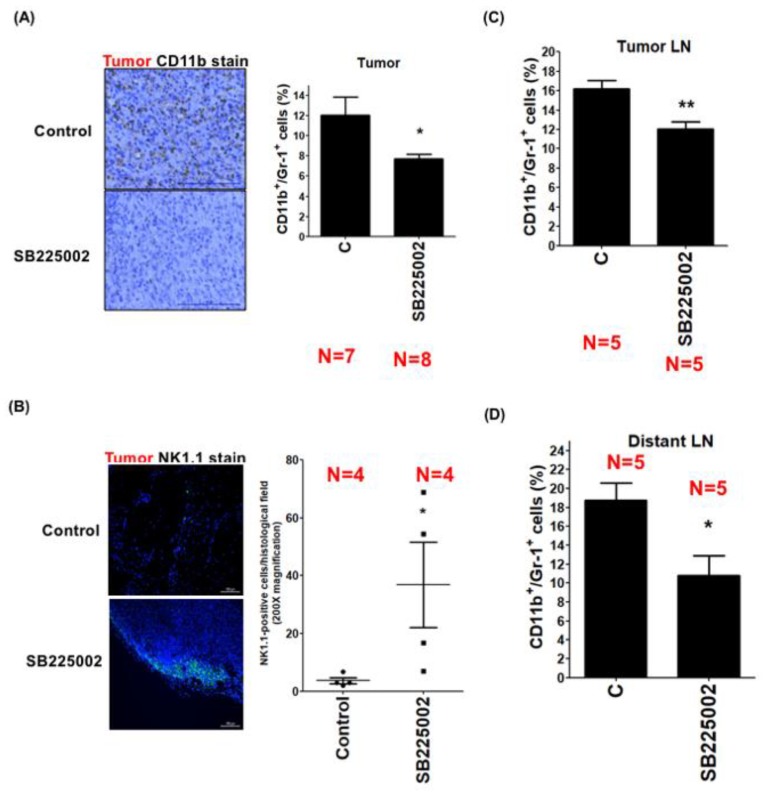
LEC-released chemokines promote myeloid-derived suppressor cell (MDSC) recruitment in the lymph nodes via CXCR2. (**A**) LC cells were injected into the mammary fat pads of nude mice. After one week, mice were treated with vehicle (control) or CXCR2 inhibitor SB225002 for another three weeks. The amount of CD11b^+^/Gr-1^+^ MDSCs in the tumors was investigated by flow cytometry. Scale bar: 50 μm. (**B**) The number of NK1.1-positive natural killer (NK) cells in the tumors was determined by immunofluorescent staining. Scale bar: 50 μm. (**C**) The amount of CD11b^+^/Gr-1^+^ MDSCs in the tumor-draining lymph nodes was investigated by flow cytometry. (**D**) The amount of CD11b^+^/Gr-1^+^ MDSCs in distant lymph nodes was investigated by flow cytometry. * *p* < 0.05; ** *p* < 0.01.

MDSCs are generated from myeloid progenitor cells in bone marrow and participate in the establishment of lymphovascular niches. The recruitment of MDSCs to tumors and lymph nodes may suppress the anti-cancer activities of immune cells. However, the key factors that mediate the recruitment of MDSCs to regional lymph nodes are still unclear. To test whether LEC-derived chemokines play a role in MDSC recruitment, we collected tumors, and tumor-draining and distant lymph nodes from mice that received vehicle or CXCR2-antagonist SB225002 treatments, and quantified the amount of CD11b^+^/Gr-1^+^ MDSCs in those tissues by flow cytometry. We found that the amount of CD11b^+^/Gr-1^+^ MDSCs in tumors was significantly reduced after SB225002 treatment (Figure 4A). These cells expressed large amounts of CXCR2 (Supplementary Figure S3). Although nude mice used in our study exhibited T-cell deficiency, they did have natural killer (NK) cells. As shown in Figure 4B, treatment of SB225002, in addition to reducing MDSC recruitment, also increased the entry of NK cells (detected by NK1.1-positive staining) into the tumors. Similar to primary tumors, the numbers of MDSCs in tumor-draining and distant lymph nodes were also significantly reduced (Figure 4C,D). To further confirm the importance of CXCR2 in MDSC recruitment, we pre-treated mice with SB225002 for two days and inoculated LC cells into the mammary fat pads of mice. Various tissues were harvested two weeks after cancer cell implantation (Supplementary Figure S4A). Similarly, tumor growth was not affected by SB225002 (Supplementary Figure S4B). However, the amounts of MDSCs in tumors, and tumor-draining and distant lymph nodes were dramatically reduced (Supplementary Figure S2C). These data suggest that LEC-derived chemokines promote the trafficking of MDSCs toward tumors and lymph nodes via CXCR2, and inhibition of CXCR2 increases the entry of NK cells, which may restore anti-cancer immunity in tumor microenvironments.

### 3.5. LEC-Derived Chemokines Upregulate Serum Amyloid A Protein in Cancer Cells to Promote Lymphatic Invasion

**Figure 5:**
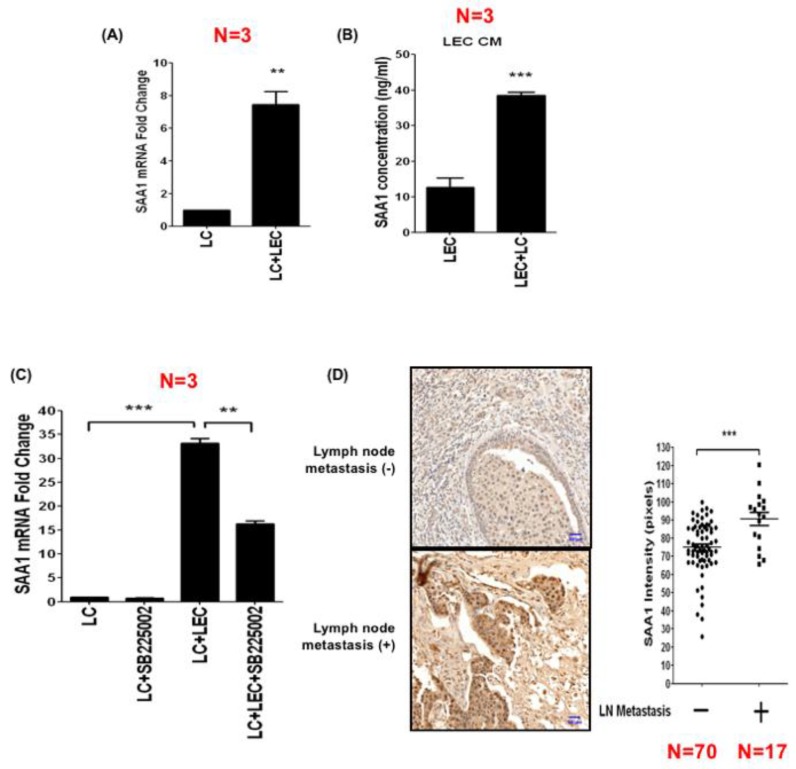
LEC-derived chemokines reciprocally upregulate SAA1 in cancer cells to promote lymphatic invasion. (**A**) The expression of SAA1 in LC cells with or without co-culture with LECs was compared. (**B**) The amount of SAA1 released from LC cells with or without co-culture with LECs was compared. (**C**) Treatment of SB225002 significantly reduced the expression of SAA1 in LC cells co-cultured with LECs, suggesting that LEC-derived chemokines stimulate SAA1 in cancer cells via CXCR2. (**D**) The expression of SAA1 in breast tumors with lymph node metastasis was significantly higher than that of breast tumors without metastasis. Scale bar: 50 μm. * *p* < 0.05; ** *p* < 0.01; *** *p* < 0.001.

We next tested whether LEC-derived chemokines could modulate gene expression in cancer cells via a reciprocal process. The gene expression profile of LC cells co-cultured with or without LECs was studied by cDNA microarray, and one of the most upregulated genes was found to be amyloid serum A 1 (SAA1). Real-time RT-PCR confirmed a four-fold increase of SAA1 in LECs co-cultured with LC cells (Figure 5A). The concentration of SAA1 in the supernatant of LC cells was elevated from 12.7 to 38.5 ng/mL after co-culturing with LECs (Figure 5B). Inhibition of CXCR2 by SB225002 attenuated the upregulation of SAA1 by LECs co-cultured with LC cells, suggesting that LEC-derived chemokines may reciprocally activate SAA1 expression in cancer cells via CXCR2 (Figure 5C). We detected the expression of SAA1 in lymph node metastasis-negative and -positive breast tumor tissues by immunohistochemical staining, and found that SAA1 levels were much higher in breast tumor tissues with lymph node metastasis (Figure 5D).

### 3.6. SAA1 Released by Cancer Cells Enhances Lymphatic Invasion by Disrupting Cell Junctions and Increasing Permeability of LECs

**Figure 6:**
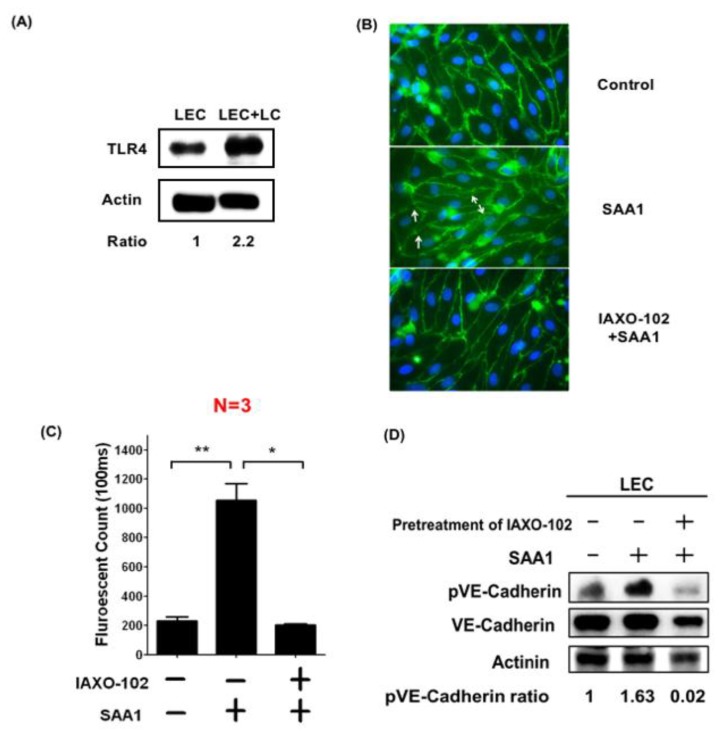
Cancer-secreted SAA1 disrupts cell junctions by inducing VE-cadherin phosphorylation via TLR4 to increase lymphatic vessel permeability. (**A**) Expression of TLR4 in LECs co-cultured with or without LC cells. (**B**) SAA1 induced dissociation of cell–cell junctions in the monolayer formed by LECs, which could be reversed by pre-treatment with a TLR4 inhibitor (IAXO-102), suggesting that SAA1 acted via TLR4 to disrupt cell junctions. (**C**) SAA1 increased permeability of the LEC monolayer, which could be suppressed by the TLR4 inhibitor. (**D**) SAA1 increased VE-cadherin phosphorylation in LECs, which could be reversed by IAXO-102. * *p* < 0.05; **, *p* < 0.01; ***, *p* < 0.001.

The mechanism by which SAA1 enhances lymphatic invasion was investigated. One major receptor for SAA1 is Toll-like receptor 4 (TLR4). We first examined the expression of TLR4 in LECs. Our data showed that TLR4 is expressed in LECs, and its expression was increased after co-culturing with LC breast cancer cells (Figure 6A). LECs were cultured on the membranes of a Transwell chamber to form an intact monolayer and were then treated with SAA1 recombinant protein in the absence or presence of TLR4 inhibitor IAXO-102, because TLR4 is expressed in LECs and is a major receptor of SAA1 [16]. As shown in Figure 6B, SAA1 induced the dissociation of cell–cell junctions in the LEC monolayer, which could be inhibited by a TLR4 inhibitor. The permeability of the LEC monolayer was examined by checking the diffusion of FITC-conjugated dextran into the lower well of the Transwell chamber. The permeability was increased in SAA1-treated LECs compared to vehicle-treated LECs, and the increase of permeability was attenuated by IAXO-102, which is consistent with the data of the immunofluorescent staining (Figure 6C). Since phosphorylation of VE-cadherin has been shown to be crucial for the regulation of cell–cell junctions [17], we analyzed the phosphorylation of VE-cadherin and found that VE-cadherin phosphorylation was enhanced by SAA1 in LECs (Figure 6D). In addition, treatment with TLR4 inhibitor IAXO-102 significantly suppressed SAA1-induced phosphorylation. Collectively, these results suggest that tumor-derived SAA1 may act via TLR4 receptor to induce the activation of p38 and JNK signaling to disrupt cell–cell junctions of lymphatic vessels, promoting lymphatic invasion.

## 4. Discussion

Although the bidirectional interaction of tumor cells and lymphatic vessels in cancer progression has been discussed previously [18], the key factors mediating the crosstalk remain exclusive. Several chemokines like interleukin-6 (IL-6) and interleukin-1b (IL-1b) have been shown to play a role in the induction of inflammation and lymphangiogenesis. For example, IL-6 signaling increased the protein synthesis of VEGF-C to promote lymphangiogenesis in oral cancer [19]. In addition, IL-6 has been found to directly stimulate the expression of VEGF-C via Src-FAK-STAT3 signaling in lymphatic endothelial cells [20,21]. Another important inflammatory mediator, IL-1b, which is released by tumor stromal cells or cancer-associated macrophages, has also been demonstrated to trigger inflammation and enhance lymphangiogenesis in tumors [22,23].

**Figure 7:**
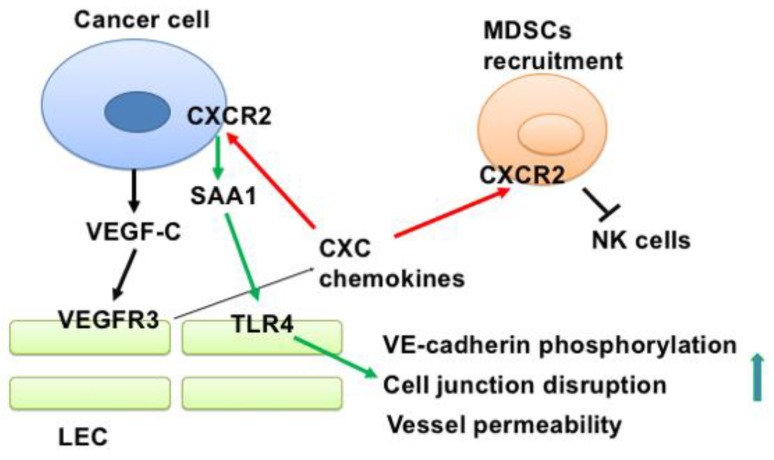
A scheme summarizes the findings of this study.

In this study, we demonstrated that cancer-derived VEGF-C stimulated the production of chemokines from LECs, and that the released chemokines in turn promoted the recruitment of MDSCs to lymph nodes to suppress anti-cancer immunity and enhanced lymphatic invasion of cancer cells by upregulating SAA1 expression (Figure 7). Several important findings are discussed asfollows. First, although VEGF-C has been shown to promote lymphangiogenesis by increasing the proliferation, migration, and tube formation of LECs, the effect of VEGF-C on global gene expression profiles in LECs is not well-characterized. We demonstrated for the first time that VEGF-C may transform naïve LECs into inflamed LECs. The most important characteristic of inflamed LECs is the upregulation of inflammatory genes, including cytokines and chemokines. Interestingly, several chemokines upregulated by VEGF-C all belong to CXCR2 ligands. This makes us hypothesize that the chemokine/CXCR2 axis may have a role in lymphatic invasion. Indeed, we showed that treatment with a CXCR2 inhibitor dramatically reduced lymph node metastasis, while primary tumor growth was not affected. Second, chemokines released from tumor-associated LECs attract CXCR2-positive MDSCs to create an immunosuppressive microenvironment. CXCR2 has been implicated in the metastasis of various cancers, including esophageal cancer, laryngeal cancer, and gastric cancer [24,25,26]. In addition, CXCL1, a cognate ligand of CXCR2, has also been reported to promote metastasis of breast cancer via CXCR2 [27]. However, whether CXCR2 plays a role in the regulation of the lymphatic microenvironment is largely unclear. A previous study demonstrated that CXCR2-expressing MDSCs are essential to promote colitis-associated tumorigenesis [28]. Additionally, disruption of CXCR2-mediated MDSC tumor trafficking enhances anti-PD1 efficacy in rhabdomyosarcoma, the most common childhood soft tissue sarcoma [29]. In breast cancer, CXCR2^+^ MDSCs have been shown to promote cancer progression by inducing the epithelial–mesenchymal transition and activated T cell exhaustion [30]. However, how MDSCs are recruited to tumor-draining and distant lymph nodes is largely unknown. We provided evidence that LEC-derived chemokines are crucial for MDSC recruitment. More importantly, inhibition of MDSC recruitment by blocking the CXCR2 signaling enhances the entry of NK cells into tumors and lymph nodes, which may restore anti-cancer immunity in vivo. Third, in addition to promoting MDSC recruitment, LEC-released chemokines also reciprocally modulate gene expression in cancer cells. We found that one of the significantly upregulated genes is SAA1. A previous study has demonstrated that breast cancer cells could release S100A4 to stimulate SAA1 and SAA3 expression via an autocrine manner, and that SAA1/SAA3 could promote metastasis of breast cancer cells [31]. In our study, we showed that the chemokine/CXCR2 axis is another key upstream activator to trigger SAA1 expression in breast cancer. Fourth, we elucidated the underlying mechanism by which SAA1 enhances lymph node metastasis. Our results demonstrate that SAA1 activates the TLR4/p38/JNK signaling pathway to enhance VE-cadherin phosphorylation and increase vessel permeability by disrupting cell–cell junctions. Our results point out the importance of SAA1 in controlling the lymphovascular microenvironment.

Since emerging evidence suggests that CXCR2 could be a druggable target in cancer therapy, the development of CXCR2 inhibitors has received much attention in recent years. Recently, the role of CXCR1/2 signaling in cancer and inflammatory diseases has been extensively reviewed [32]. Although potent CXCR2 antagonists have been reported, most of the antagonists under clinical trials were tested in non-cancerous diseases like asthma, airway inflammation, and coronary heart diseases [33,34,35]. Therefore, the application of CXCR2-targeted cancer treatment awaits further studies.

Taken together, we conclude that the crosstalk between cancer cells and LECs via VEGF-C/VEGFR3 and chemokine/CXCR2 signaling plays a pivotal role for the establishment of lymphovascular niches, and CXCR2 may be a target for the inhibition of lymphatic metastasis and for the re-activation of anti-cancer immunity in breast cancer.

## 5. Conclusions

In summary, we elucidate how breast cancer cells with high lymphatic tropism produce VEGF-C to upregulate the expression of CXC chemokines in LECs, and how the chemokines released from LECs promote lymphatic invasion and MDSC recruitment to increase tumor metastasis and to attenuate anti-cancer immunity. Our results provide a new strategy to inhibit breast cancer metastasis by modulating the lymph node microenvironment.

## Supplementary Materials

The following are available online at https://www.mdpi.com/2072-6694/11/8/1120/s1, Figure S1: Treatment of the conditioned medium of LC cancer cells upregulated the expression of inflammatory genes, chemokines and cytokines in LECs; Figure S2: Expression of CXCR2 on the surface of MDA-MB-231 and LC breast cancer cells detected by flow cytometry; Figure S3: Expression of CXCR2 on the surface of CD11b^+^/Gr-1^+^ MDSCs detected by flow cytometry; Figure S4: CXCR2 inhibitor prevents MDSC recruitment to tumors, tumor-draining lymph nodes (TDLN) and distant lymph nodes. (A) SB225002 was injected 48 h before the inoculation of cancer cells into the mammary fat pads of nude mice. Tumors, spleens, and lymph nodes were harvested for analysis two weeks later. (B) The tumors weight of the control and SB225002-treated mice were not significantly different. (C) The amount of CD11b^+^/Gr-1^+^ MDSCs in tumor, spleens and lymph nodes was investigated by flow cytometry.
