# Supplementary material for: Cancer-Derived VEGF-C Increases Chemokine Production in Lymphatic Endothelial Cells to Promote CXCR2-Dependent Cancer Invasion and MDSC Recruitment
Source: Cancers (Basel). 2019 Aug 6;11(8):1120. doi: 10.3390/cancers11081120 (PMC6721484; doi:10.3390/cancers11081120)
Supplement: Supplementary file 1 [file cancers-11-01120-s001.pdf]

Supplementary Material: Cancer-Derived VEGF-C Increases Chemokine Production in Lymphatic Endothelial Cells to Promote CXCR2-Dependent Cancer Invasion and MDSC Recruitment

Jing-Yi Chen, You-Syuan Lai, Pei-Yi Chu, Shih-Hsuan Chan, Lu-Hai Wang and Wen-Chun Hung

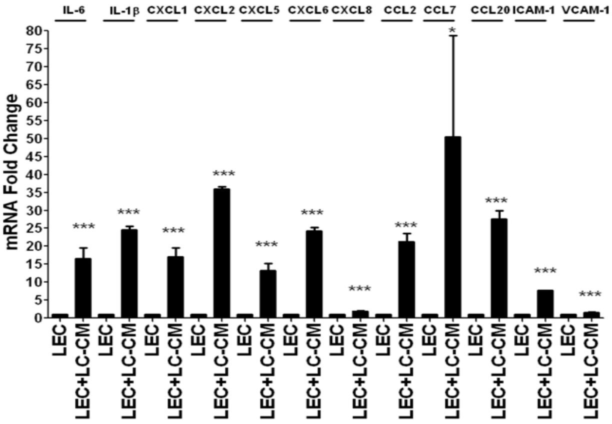

Figure S1. Treatment of the conditioned medium of LC cancer cells upregulated the expression of inflammatory genes, chemokines and cytokines in LECs.

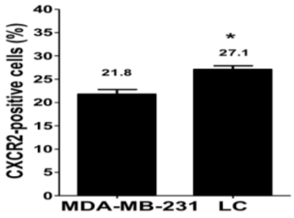

Figure S2. Expression of CXCR2 on the surface of MDA-MB-231 and LC breast cancer cells detected by flow cytometry.

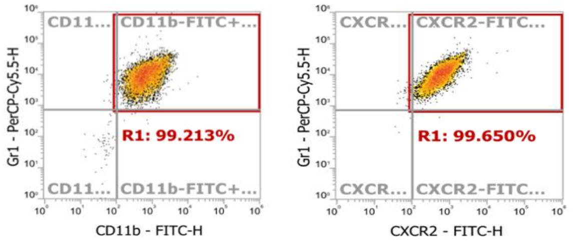

Figure S3. Expression of CXCR2 on the surface of $CD11b^{+}/Gr\text{-}1^{+}$ MDSCs detected by flow cytometry.

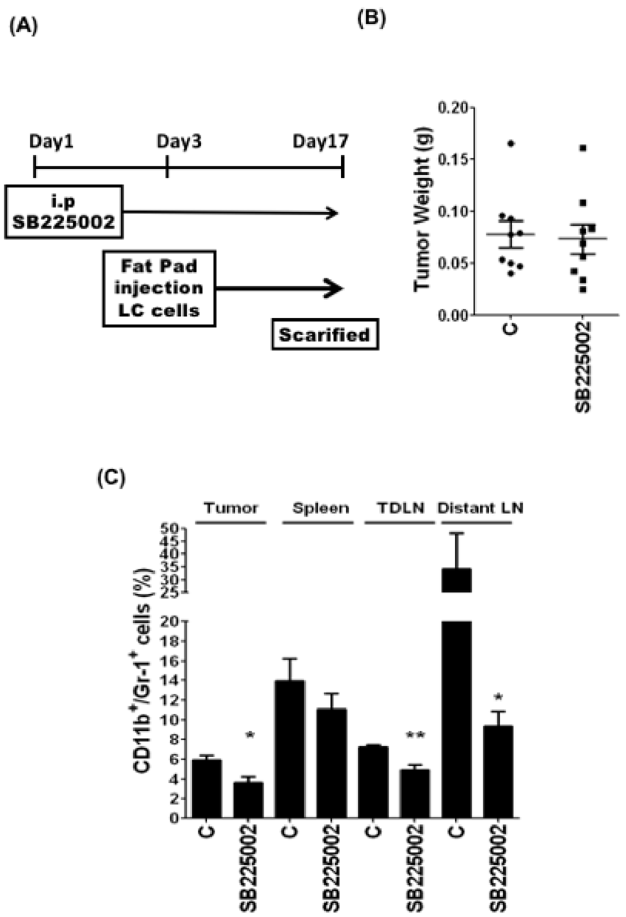

Figure S4. CXCR2 inhibitor prevents MDSC recruitment to tumors, tumor-draining lymph nodes (TDLN) and distant lymph nodes. (**A**) SB225002 was injected 48 h before the inoculation of cancer cells into the mammary fat pads of nude mice. Tumors, spleens, and lymph nodes were harvested for analysis two weeks later. (**B**) The tumors weight of the control and SB225002-treated mice were not significantly different. (**C**) The amount of $CD11b^{+}/Gr\text{-}1^{+}$ MDSCs in tumor, spleens and lymph nodes was investigated by flow cytometry.
